# Autobiographical memories of interpersonal trust in borderline personality disorder

**DOI:** 10.1186/s40479-020-00130-w

**Published:** 2020-08-03

**Authors:** Janina Botsford, Babette Renneberg

**Affiliations:** Clinical Psychology and Psychotherapy, Freie Universität Berlin, Habelschwerdter Allee 45, Raum JK 24/215, 14195 Berlin, Germany

**Keywords:** Borderline personality disorder, Interpersonal trust, Autobiographical memories, Content analyses

## Abstract

**Background:**

Establishing and maintaining interpersonal trust is often difficult for patients with Borderline Personality Disorder (BPD). How we trust is influenced by prior trust experiences.

**Methods:**

For the investigation of trust experiences, autobiographical memories of *n* = 36 patients with BPD and *n* = 99 non-clinical controls were examined. Trust objects and interaction partners, emotional valence, perceived relevance and memory specificity were analyzed.

**Results:**

Content analyses revealed that patients with BPD recalled mostly situations in which their trust was failed by family members or romantic partners. In addition, patients with BPD considered memories with trust and mistrust more relevant for their current lives than the control group. Our results correspond with findings that BPD patients have difficulties trusting close others as well as with theoretical assumptions about deficits in mentalizing and epistemic trust in patients with BPD.

**Conclusion:**

In conclusion, our findings should encourage clinical practitioners to address trust deficits towards close others, as well as omniscient negative memory retrieval and interpretation biases which might influence current trust behavior.

## Introduction

“Trust is essential to initiate, establish and maintain social relationships.” This quote from Balliet and Van Lange [1] illustrates the importance of interpersonal trust for the development and maintenance of beneficial social relationships. Borderline Personality Disorder (BPD) is characterized by marked difficulties in interpersonal functioning [2]. Prior research shows that patients with BPD display alterations in interpersonal trust [3]. An extensive search of the literature on the definition of trust revealed a variety of different conceptualizations. This variety of conceptualizations can be broadly clustered into attitudinal [4] and behavioral [5] conceptualizations of interpersonal trust. In our research, we have adopted the behavioral perspective on interpersonal trust. In the behavioral perspective, cognitions, emotions and behavioral tendencies are understood as prerequisites of interpersonal trust. Because these aspects are considered, the behavioral perspective seems like the most comprehensive conceptualization to us. We follow the definition of Thielmann and Hilbig [6] who understand interpersonal trust as: “A risky choice of making oneself dependent on the actions of another in a situation of uncertainty, based upon some expectation of whether the other will act in a benevolent fashion despite an opportunity to betray.”. In a recent study by our research group [7], we found that patients with BPD do not seem to display alterations in interpersonal trust in all facets of the construct of trust. As our research is based on a behavioral understanding of interpersonal trust, different facets of trust are understood as different kinds of trustful acts, i.e. entrusting material items (e.g. lending one’s harddrive to a friend) versus entrusting one’s well-being (e.g. driving in a car with a stranger). In our previous study, we compared interpersonal trust behavior measured by a newly developed scenario-based trust questionnaire (Interpersonal Trust Scenario Questionnaire – ITSQ) from patients with BPD, patients with Major Depressive Disorder (MDD), patients with Social Anxiety Disorder (SAD) and non-clinical control participants. Our results suggested that patients with BPD overall reported least trusting behavior compared to all other groups. In detail, patients with BPD reported significantly less trusting behavior when entrusting material items to people they know such as friends or partners than non-clinical control participants and patients with MDD. However, when entrusting their well-being to unknown people, patients with BPD did not report less trusting behavior than the other groups.

In mentalization-based therapy (MBT) the construct of epistemic trust has gained much attention. The capacity to mentalize has received most attention in patients with BPD [8] as these patients seem to display poor mentalization skills and are characterized by epistemic mistrust [8, 9]. Epistemic trust differs from interpersonal trust: Interpersonal trust generally refers to all kinds of trust objects (e.g., personal information or worthy items) in situations with different interaction partners, while epistemic trust describes and focusses on “openness to the reception of social communication that is personally relevant and of generalizable significance” [10]. Epistemic trust usually occurs for the first time in early social learning environments and early attachment experiences [9]. It is supposed to influence the ability to mentalize, indicating the ability to recognize and correctly name mental states and to use this ability in a flexible way and as a reliable source of information for choices and behaviors [11]. Even though the two trust concepts are not equal, they do seem to share content. Epistemic trust could be understood as interpersonal trust towards a person who is transmitting personally relevant information.

Different studies suggest that trust experiences influence a person’s trust behavior to a large degree [6, 12–15]. To learn about trust experiences of patients with BPD and to deepen our understanding of trust alterations in BPD, we investigated autobiographical memories (ABMs) about trust.

Autobiographical memories (ABMs) are personal memories about events an individual has experienced and are therefore always self-referential. ABMs hold an identity-establishing function [16] because by integrating memories from the past, meaningful narratives about the self are established [17]. In his book “Searching for memory: the brain, the mind and the past” Schacter [18] emphasizes the importance of the ABMs for the self. Functions of the self, such as problem solving, mood regulation, and social interaction are based on ABMs [19] as experience-based information can be used as a reference point for current situations [20]. One clinically relevant feature of ABMs is the specificity of the memory [19]. The idea is that memories are restored on different levels of specificity: for instance, lifetime periods: “During my childhood I had a pet” versus general events: “During the last year of my marriage, my husband and I were fighting a lot” versus event-specific knowledge: “The last dinner we ate together before we separated was salmon, spinach and potatoes. It tasted great.”. Studies on ABMs in the context of mental disorders primarily investigated this feature of the retrieved memories [19–21]. In patients with Major Depression (MDD), overgeneralized autobiographic memories (OGM) are a robust finding [19, 22], whereas in patients with BPD research results on specificity are inconsistent. Bech, Elkilit and Simonsen [20] in their systematic review reported no OGM in BPD when comorbid depression was controlled for. Beran, Richman and Unoka [23] on the other hand found a large effect size for OGM in BPD. One explanation for the diverging results could be that Beran et al. [23] did not control for comorbid MDD. One possible interpretation of the differences of retrieval style between patients with MDD and patients with BPD is provided by Conway and Pleydell-Pearce’s model [24] about ABMs and the working self. The idea is that each individual has a set of different self-schemata that are connected to specific goals. When a person wants to pass a test in college for example, the self-schema of the successful academic individual might be activated. The working self/activated self-schema subsequently determines from which level of specificity information can be recalled. According to this theory, an individual with the goal of wanting to pass a test should be able to recall relatively specific information (e.g., “On page 4 of the biology book the process of cell division was explained. Cell division works as follows: ...”). An individual with the goal to relax, however, would be able to recall rather generic information (e.g., “During our last holiday the kids were watching TV most of the time.”). It may be that in research settings patients with BPD activate a performance oriented working self and want “to do their best”. Subsequently, they are able to recall relatively specific information. Patients with MDD on the other hand often suffer from a ruminative cognitive style, often show deficits in executive functioning and may have difficulties motivating themselves, which makes it more difficult for them to activate a performance oriented working self. Besides the retrieval style, another important characteristic of ABMs of patients with BPD is the emotional valence. Patients with BPD seem to recall more negative life events than non-clinical controls [25–30] pointing towards a bias for negative memory retrieval and the prevalence of a negative view of self and others [29]. Rosenbach and Renneberg [30] additionally reported that patients with BPD considered their ABMs about rejection as more relevant for their current lives than non-clinical controls, which would speak for the heightened sensitivity and relevance towards this topic.

For a comprehensive understanding of trust memories and alterations in trust behavior, it is interesting to investigate also the content of ABMs. So far, only one published study examined thematic content of memories of patients with BPD. Guruprasad and Bohla [31] examined themes and structure of self-narratives from five patients with BPD to explore their history of psychological difficulties. The themes of the memories were agency (themes of power, achievement, mastery, independence, autonomy, separation), communion (themes of relationship, connection, intimacy, nurturance, helping, closeness), redemption (negative events that begin with struggles, obstacles, and setbacks, but end with moments of triumph, growth, rejuvenation, and positive emotion) and contamination (sequences begin with hope or positive circumstances and end in frustration, disappointment, and dejection). Results suggested that the narratives tended to be generic in nature, were not well-integrated within the larger self-concept and contained predominantly “contamination” themes. These results correspond with findings about disrupted self-concepts as well as with experiences of abuse, neglect and lack of support, which are all characteristic of BPD [32].

To our knowledge, there are no studies on ABMs of trust in BPD yet. However, for a deeper understanding of alterations in trust behavior in BPD, it seems necessary to examine trust experiences. Having in mind that patients with BPD do not seem to display trust alterations on all facets of interpersonal trust (i.e., only when entrusting material items to people known to them; see [7]) we wanted to examine if those features were reflected within their ABMs of trust, too. The first aim of the present study was to investigate ABMs of trust regarding the interaction partners and trust objects in both patients with BPD and non-clinical controls. Our second aim was to compare interaction partners and trust objects between patients with BPD and non-clinical controls. Our third aim was to investigate whether alterations in ABMs in BPD – namely the negative emotional valence of memories, specificity, and today’s relevance of the retrieved memories – can be found in ABMs of trust. We hypothesized that patients with BPD recall more negative trust situations, consider their ABMs of trust as more relevant to their current lives and do not display more generalized ABMs than non-clinical controls. The first two aims were examined in an exploratory way.

## Methods

### Participants

*N* = 36 patients with Borderline Personality Disorder (30 female, 6 male, *M*_*age*_ = 29.6, *SD*_*age*_ = 9.9) participated in our study, of whom five (13,9%) had both a comorbid MDD and PTSD, seven (33.3%) had a comorbid MDD only and one (16,7%) had a comorbid PTSD only. All patients were diagnosed using the Structured Clinical Interview for DSM-IV Axis-I and Axis-II [33, 34]. Furthermore, *n* = 99 non-clinical controls (70 female, 29 male, *M*_*age*_ = 39.1, *SD*_*age*_ = 17.9) participated. Patients with BPD were recruited at a borderline-specific inpatient treatment facility. Non-clinical controls were recruited in different settings: the majority of non-clinical control participants was recruited at a public event (Lange Nacht der Wissenschaften / Long Night of the Sciences) at Freie Universität Berlin, a smaller part (*n* = 15) was recruited via emails and postings in internet forums and on social media platforms. Eligible for the study were people over 18 years of age with sufficient knowledge of the German language to understand the questionnaires. Current mood in non-clinical controls was assessed via the 5-item World Health Organization Well-Being Index (WHO-5) [35]. Control participants reported current mental well-being scores of M = 16.19 (SD = 3.07) indicating good overall well-being. Ten participants reported scores from 12 to 10, which indicate a mild depressive mood in those ten participants (cut-off for mild depressive symptoms < 13). The ethics committee of Freie Universität Berlin (No. 182/2018) approved the study protocol.

### Measures

#### Well-being index

The WHO-5 is a short self-report measure for the assessment of well-being and at the same time serves as a screening instrument for current depressive symptoms. The measure has good construct validity as a unidimensional scale measuring well-being [35].

#### Questionnaire for the assessment of ABMs

In the self-report questionnaire, participants were asked to describe two memories of interpersonal trust situations. For situation 1, participants were asked to describe a situation in which they actually showed trust (e.g., “My housemate proposed that he/she could take care of my dog while I am away and I let him/her do it”.). For situation 2, participants were asked to describe a situation in which trust could have been shown but was not (e.g., “A friend asked me to buy a concert ticket for him/her and said that he/she would reimburse me as soon as possible. I did not do it, because I know from previous situations that the friend is unreliable and probably would not give me the money”). Furthermore, participants were instructed to write down their emotions related to these situations, how important the situation is for their current life on a scale from 1 (not relevant at all) to 5 (very relevant) and indicate for the first situation whether their trust was failed or not.

### Procedures

Of the complete sample (*n* = 135), most participants (*n* = 120) completed a paper-pencil version of the questionnaire. Only a small number of the control group (*n* = 15) participated online. The groups (online vs paper-pencil version) did not differ significantly from each other in age, gender, and length of the texts. First, basic demographic information (age and gender) was completed, followed by the WHO-5 and the questions on ABMs, associated emotions, relevance and – for situation 1 – whether trust was failed or not. Two trained research assistants transcribed all handwritten situation descriptions in Microsoft Excel. Spelling errors were not corrected as we analyzed content only (in opposite to linguistic patterns for example). Text analysis was run in MaxQDA and the analysis was conducted based on the qualitative text analysis approach by Mayring [36].

### Development of the category system

Category systems for trust objects and interaction partners were developed. Deductive categories for trust objects were derived using facets of published questionnaires (*n* = 6) on interpersonal trust [4, 37–46]. After the facets were controlled for overlapping content, the following categories were identified: general trust, trust in a person’s benevolence, trust in a person’s dependability, trust in a person’s reliability, trust in a person’s honesty, and trust in a person’s competency. Definitions and examples for those categories can be found in Table 1.

**Table 1:** Category definitions and examples

Category	Definition	Example
General trust	This category describes a general ability and willingness to trust.	*“In fact, trust is a basic compass in my life and determines most of my actions.”*
Trust into a person’s benevolence	This category describes situations in which both the physical and emotional well-being are regarded as the trust object.	*“One night I was by myself in the middle of the city after a fight with someone. I did not know how to get home and I wasn’t feeling well. A cab driver pulled over and asked me if he could drive me somewhere. I was not sure whether this was a good idea, however, I agreed and got into the car. The driver was very kind and dropped me off at home. Even though I was unsure, I trusted him blindly.”*
Trust into a person’s dependability	This category describes situations that involve a practical component of trust, such as adherence to organizational agreements.	*“Vacation in a secluded area with my husband. He promised to pick me up later to go back to our cabin together. It was dawning by the time he came back. I trusted him and waited patiently for his return.”*
Trust into a person’s emotional reliability	This category describes situations in which one trusts into the empathic reflection and understanding of another.	*“In times of crisis (mainly due to partners/relationships) I was able to tell my little sister all my problems and found comfort and security in her support (from the age of 19 years).”*
Trust into a person’s competency	This category describes situations in which one trusts into the skills and abilities of another.	*“Birth of first child in hospital in 2010: My midwife was with me. I got to know her in preparatory courses. Her presence made the birth much easier for me, as I was able to trust into her professional skills.”*
Trust into a person’s honesty	This category describes situations in which one trusts in the honest and sincere statements of another.	*“My boyfriend at the time told me he’d changed and that he wanted the relationship to work. That was the third chance I’d given him after two previous breakups and this time what he said was true.”*

Two independent evaluators, who were trained to a minimum of 80% agreement, assigned the text material to the relevant categories using a detailed coding manual [47]. Interaction-partner categories were inductively formed during the coding process. The following interaction-partner categories were derived: family, friends, romantic partners, colleagues, healthcare professionals and strangers. During the coding process, more specific subcategories were developed. For example, for the category trust into a person’s competency, subcategories like trust into a person’s competency during counseling were developed. Almost all subcategories could be applied to both situations with one exception: the subcategory “instruction misunderstood” was developed for situation 2 only. This subcategory had to be developed because some participants misunderstood the instruction (“Please describe a situation in which you could have shown trust, but after all you did not.”) and described a situation, in which indeed they did show trust, but the trust was failed by the interaction partner. For both category systems, a number of decision rules were established before and during the rater training. For example, if a sentence contained two different statements, which clearly represented different trust contents, the sentence was separated and each part coded according to the fitting category (e.g., “Trust at home with the family. One can rely on the others and have trust that secrets will not be told to other people and that the others will tell the truth.” – Codes: emotional reliability and honesty). If the two raters disagreed, a short discussion took place and a decision on the category was made. If discrepancies could not be resolved, the statement was coded “irrelevant”. In both cases, the statement was counted as “no agreement” in the calculation of Cohen’s kappa. After the described training, the two coders reached a good interrater agreement of κ = .80.

### Ratings of emotional valence and specificity

Valences of emotions assigned to the trust situations were categorized as positive, negative or mixed (e.g., sad and relieved). Agreement between raters *κ* = .94 was very good.

Specificity of memories was categorized as specific (e.g., “In 2013 when I traveled through South America with my best friend, we had a car accident. I called my brother, asked him to send me money, and begged that he would not tell my parents. He kept my secret and sent me the money.”), categorical (e.g., “The times I have talked to my social worker about my anxiety, I trusted that she would not tell anybody else.”) or extended (e.g., “In general, I find trust in marriages extremely important, nevertheless, I don’t trust and I have never trusted.”). Very good agreement of *κ* = .89 was reached.

### Statistical analysis

Frequencies and percentages of trust objects and interaction partners were determined, first for overall participants and then for each group separately. Relationships between group (patients with BPD vs. non-clinical controls) and trust objects, interaction partners, valence of emotions, specificity and whether the trust in situation 1 was failed or not were conducted using Chi Square Tests of Independence because data were categorical. Last, comparison of the relevance of the described ABMs was conducted using non-paired t-tests.

## Results

Our main focus was to analyze the content of trust memories of patients with BPD and non-clinical control participants. To provide a comprehensive picture of our results, we also report frequencies of named trust objects and interaction partners as well as the emotional valence, specificity and perceived relevance of the reported trust memories.

### Trust objects

#### Situation 1: A situation in which trust was shown

**Fig. 1:**
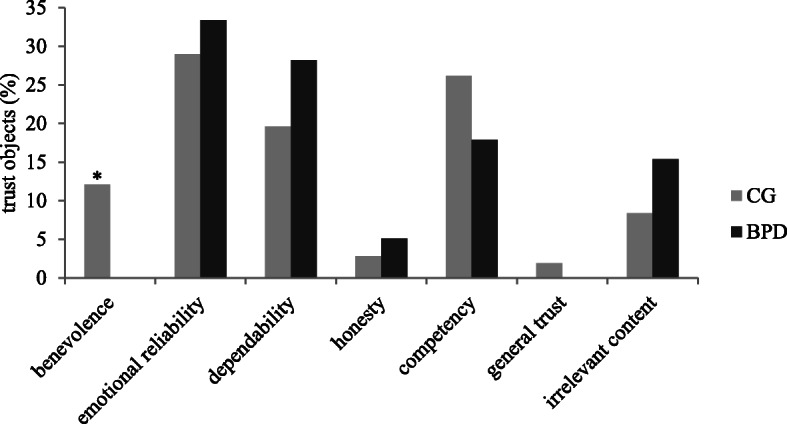
Frequencies (percentage of all recalled memories) of trust objects described in situation 1. BPD = patients with borderline personality disorder; CG = non-clinical control participants

Frequencies (in percentage of all recalled memories) of trust objects are presented in Fig. 1. Patients with BPD did not report memories of trusting into another person’s benevolence, thus a significant difference to the control group was found *χ* 2(1) = 5.23, *p* = .02, φ = −.19. All other comparisons were non-significant (*p* > .05).

#### Situation 2: A situation in which trust could have been shown but was not

**Fig. 2:**
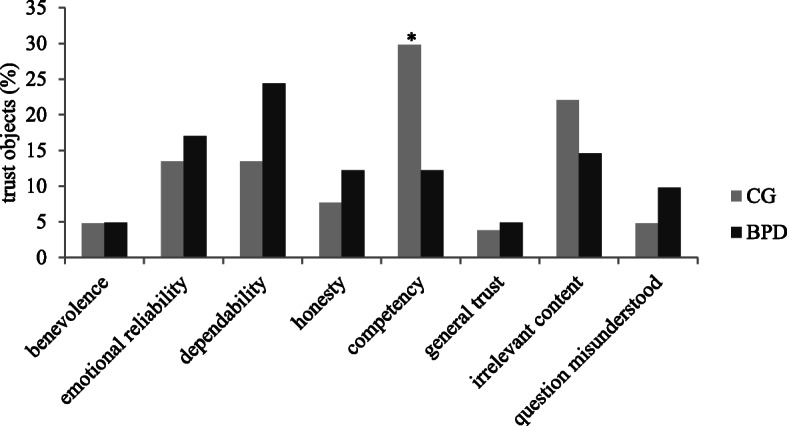
Frequencies (in percentage of all recalled memories) of trust objects in situation 2. BPD = patients with borderline personality disorder; CG = non-clinical control participants

Frequencies (in percentage of all recalled memories) of trust objects are presented in Fig. 2. Patients with BPD reported significantly fewer memories about failing to trust into someone’s competency than non-clinical controls *χ*^*2*^(1) =4.10, *p* = .04, φ = −.17. All other comparisons were non-significant (*p* > .05).

### Interaction partners

#### Situation 1: a situation in which trust was shown

**Fig. 3:**
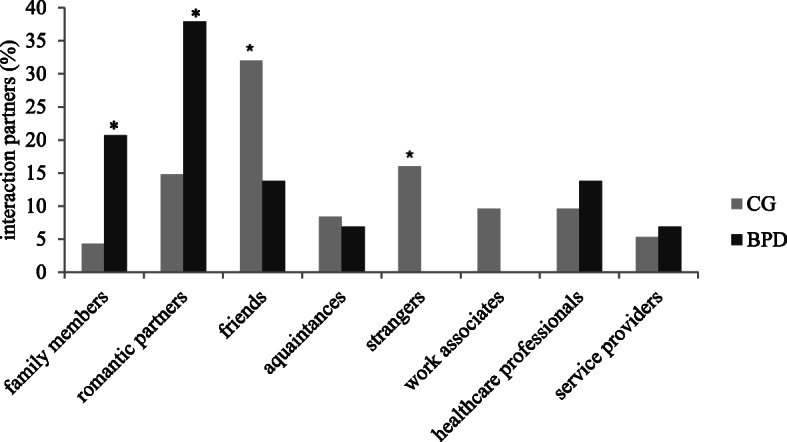
Frequencies (percentage of all recalled memories) of interaction partners described in situation 1. BPD = patients with borderline personality disorder; CG = non-clinical control participants

Frequencies (in percentage of all recalled memories) of interaction partners are presented in Fig. 3. Compared to control participants, patients with BPD reported significantly more trust memories with family members *χ*^*2*^(1) = 6.14, *p* = .01, φ = .21 and romantic partners *χ*^*2*^(1) = 4.71, *p* = .03, φ = .18 whereas non-clinical controls reported significantly more trust memories with friends *χ*^*2*^(1) =5.16, *p* = .02, φ = −.19 and strangers *χ*^*2*^(1) = 6.14, *p* = .01, φ = −.21 as interaction partners. All other comparisons were non-significant (*p* > .05).

#### Situation 2: A situation in which trust could have been shown but was not

**Fig. 4:**
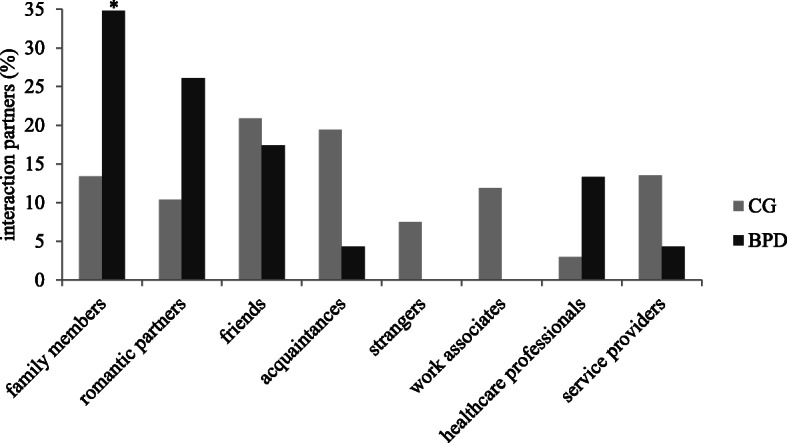
Frequencies (in percentage of all recalled memories) of interaction partners in situation 2. BPD = patients with borderline personality disorder; CG = non-clinical control participants

Frequencies (in percentage of all recalled memories) of interaction partners are presented in Fig. 4. Patients with BPD reported significantly more memories about failing to trust into family members than non-clinical controls *χ*^*2*^(1) = 4.14, *p* = .04, φ = .18. All other comparisons were non-significant (*p* > .05), however, the differences within the categories of romantic partners and strangers followed the same pattern like in situation 1 – a situation in which trust was shown. This means that patients with BPD reported more situations with romantic partners than non-clinical controls, and non-clinical controls reported more situations with strangers than patients with BPD.

### Emotional valence

#### Situation 1: A situation in which trust was shown

Patients with BPD reported more negative and mixed emotions in situations where they showed trust, whereas non-clinical controls reported more positive emotions for situations in which they showed trust (CG vs. BPD percentages of positive emotions: 65.6% vs. 36.7%; negative emotions: 30.0% vs. 50.0%; mixed emotions: 4.4% vs. 13.3%). The overall difference was significant χ 2(1) = 8.46, *p* = .015, φ = .27. Post-hoc tests revealed that patients with BPD reported significantly less positive emotions χ 2(1) = 7.67, *p* = .01, φ = −.25 compared to non-clinical controls. Both other comparisons were non-significant (*p* > .05).

#### Situation 2: A situation in which trust could have been shown but was not

Both groups rated the emotional valence of memories of situations in which they did not show trust as negative. Non-clinical controls and patients with BPD did not differ significantly from each other (*p* > .05; CG vs. BPD percentages of positive emotions: 7.9% vs. 0.0%; negative emotions: 90.8% vs. 100.0%; extended memories: 1.3% vs. 0.0%).

### Trust situation outcome

#### Situation 1: A situation in which trust was shown

Patients with BPD reported significantly more often that their trust was failed (36.7%) compared to non-clinical controls (4.4%;) χ 2(1) = 21.36, *p* < .001, φ = .42.

### Specificity

#### Situation 1: A situation in which trust was shown

The specificity of memories of situations in which trust was shown did not differ between the groups (*p* > .05; CG vs. BPD percentages of specific memories: 67.8% vs. 60.0%; categorical memories: 13.3% vs. 20.0%; extended memories: 18.9% vs. 20.0%).

#### Situation 2: A situation in which trust could have been shown but was not

The specificity of memories of situations in which trust was not shown did not differ between the groups (*p* > .05; CG vs. BPD with MDD vs. BPD without MDD percentages of specific memories: 71.1% vs. 56.7%; categorical memories: 17.1% vs. 33.3%; extended memories: 11.8% vs. 10.0%).

### Relevance

#### Situation 1: A situation in which trust was shown

Patients with BPD rated their memories of situations in which they showed trust as significantly more relevant (*M* = 4.57, *SD* = 0.77) for their current lives than non-clinical controls (*M* = 3.64, *SD* = 1.25) *t* = − 3.81, *p* < .001, d =. -.89.

#### Situation 2: A situation in which trust could have been shown but was not

Patients with BPD rated their memories of situations in which they did not show trust as significantly more relevant (*M* = 3.91, *SD* = 1.28) for their current lives than non-clinical controls (*M* = 3.13, *SD* = 1.26) *t* = − 2.66, *p* = .01, d = −.62.

## Discussion

The purpose of the current study was to provide insight into autobiographical memories (ABMs) of trust in patients with BPD compared to non-clinical controls. To our knowledge, our study is the first to provide information about ABMs of trust in patients with BPD.

The main result suggests that patients with BPD, when remembering trust, mainly recall memories in which their trust was failed by family members or romantic partners. Interestingly, they also recalled memories where they themselves failed to trust their family members. This result corresponds with findings on self-reported difficulties in trusting close others by patients with BPD [7].

The main focus of the current study was to analyze content of trust memories of patients with BPD and non-clinical control participants. To provide a comprehensive picture of such memories, we additionally reported frequencies of named trust objects (e.g. emotional reliability) and interaction partners as well as emotional valence, specificity and perceived relevance of trust memories.

The first aim of our study was to provide insight into what and about whom patients with BPD and non-clinical controls trusted. Current results suggest that especially trust into a person’s emotional reliability (30.1%) followed by trust into a person’s dependability (21.9%) and competency (24.0%) were the major trust objects named. Similarly, competency (14.4), dependability (16.6), and emotional reliability (14.4) were also the most frequently named categories in trust situations in which individuals did not show trust (situation 2). Participants of both groups reported most often to trust friends (27.6%) and romantic partners (20.3%). Besides this, they reported most often friends (20.0%), family (18.9%), acquaintances (15.5%) and romantic partners (14.4%) in situations in which they could have trusted but did not (situation 2). Emotional factors like empathetic listening and practical aspects like trusting into each other’s competencies make trust one of the most important factors for beneficial human relationships.

Our second aim was to examine differences in trust objects and interaction partners between patients with BPD and non-clinical controls. Patients with BPD did not report memories of trust into a person’s benevolence at all, while non-clinical controls did. This difference was significant.

Interestingly, in both groups, participants’ trust into a person’s benevolence was always related to unknown interaction partners. One explanation for this might be that benevolence (the entrusted interaction partner does not want to harm the trusting person’s emotional and physical well-being) is something that is usually expected as given in relationships with close others, while in interactions with unknown people one does not have any information about the entrusted person’s intentions. Patients with BPD do not seem to expect the benevolence of close others as given, however.

Patients with BPD showed a pattern to remember family members and romantic partners as interaction partners more often than non-clinical controls, while non-clinical controls remembered friends and strangers more often than patients with BPD. These differences were significant. In fact, patients with BPD did not remember any situation at all in which strangers were their interaction partners, which corresponds with the above described result on trust into someone’s benevolence. The result that patients with BPD remembered mostly situations with family members and romantic partners corresponds with the results from a study about memories of rejection [30]. In a linguistic analysis of ABMs of rejection, patients with BPD remembered rejection by family members significantly more often than patients with MDD and healthy controls. Besides this, in our study patients with BPD indicated significantly more often than non-clinical controls that their trust was failed. This result corresponds with results from Guruprasad and Bohla [31] who found that participants described mostly situations that started with hope and optimism and ended with a failure of those positive expectations.

In the current study, an illustrative example of such a situation from a patient with BPD was:

“My car was broken and standing in front of my house. I had to go to the hospital and could not take care of it, so my dad promised to me to bring it to the garage. When I returned from the hospital, the car was still standing in the same place and my father obviously again did not stick to his word.”

It is striking that the patient who described this memory seems to have frequently experienced similar situations (my father obviously again did not stick to his word). As trust behavior is influenced by trust experiences [6], it does not seem surprising at all that patients with BPD reported difficulties trusting close others [7] Conway and Pleydell-Pearce’s [24] model about ABMs provides possible explanations for how trust experiences might influence current trust behavior. As described in the introduction, the model assumes that individuals contain sets of different self-schemata that are connected to specific goals. A person who has made repeated negative trust experiences with close others might develop the self-schema of “the betrayed one” with the goal to not be betrayed again. This goal could be attained by not showing trust towards close others in the first place.

The two groups differed in situations in which trust could have been shown but was not (situation 2). Patients with BPD recalled fewer memories about failing to trust into someone’s competency than non-clinical controls. One explanation for this finding could be that patients generally experience more situations in which they have to trust in a person’s competency, such as those in psychotherapy for example:

“During sessions with my therapist. I trust that we can take breaks whenever I want to. That we can do things without any pressure. That I can even say negative things out loud and still I will not be abandoned. That I am even allowed to scream. That he will not blame me, even if I will not accomplish my goals. That he will approach me. That there is not so much I can do wrong.”

Besides, in situations in which trust could have been shown but was not (situation 2), differences within interaction partner categories followed a similar pattern like in situation 1 – a situation in which trust was shown: here too, patients with BPD named family members and romantic partners as interaction partners more often than the control participants did. The difference within the category of family members was significant.

This result supports the idea that patients with BPD develop difficulties in trusting family members already early in life. Besides, this corresponds with research results indicating a high amount of childhood maltreatment in BPD [32, 48, 49] and the assumptions by Fonagy and colleagues that epistemic mistrust in BPD may be rooted in dysfunctional early attachment experiences [10].

The third aim of our study was to investigate whether alterations in ABMs from patients with BPD - for example, emotional valence of memories - can be found in ABMs of trust. In line with our hypotheses, patients with BPD related their ABMs of trust mostly to negative emotions and considered those memories as more relevant for their current lives than non-clinical controls. Prior studies on ABMs of patients with BPD found a tendency to recall mostly negatively valenced memories [25–30]. Bech, Elklit and Simonsen [20] argue that this tendency could be explained by a higher amount of negative life experiences in comparison to non-clinical controls. Another explanation is provided by Renneberg et al. [29], who state that this tendency could also reflect the extremely negative view patients with BPD have of themselves, other people, and the world in general.

Concerning relevance ratings, our results correspond with the results from Rosenbach and Renneberg [30] who also found that patients with BPD rated their ABMs of rejection as more relevant for their current lives than non-clinical controls. These results support the idea that current difficulties with trust or rejection are strongly influenced by prior negative experiences and speak for a heightened sensitivity towards these topics. Regarding the specificity of memories, our results suggest that patients with BPD do not display OGM when remembering trust, even when not controlling for comorbid depression. This result corresponds with findings by a majority of studies concerning OGM in BPD [20].

Limitations of the current study are that generalization of the current results is limited due to the small number of male patients in our study. Furthermore, BPD symptomatology was not assessed in the control group, as our assessment took place in a public and anonymous setting in which task duration was limited, which made it difficult to assess more sensitive information.

Current results emphasize the role of family members and romantic partners within memories of trust in patients with BPD. More specifically, patients with BPD mainly recall trust situations in which their trust was failed by family members and romantic partners. This is highly relevant for the current lives of the patients, as indicated by relevance ratings. Consequently, patients with BPD report mostly negative emotions when recalling ABMs of trust.

## Conclusions

Taken together, our study is the first to provide insights into the nature of ABMs of trust from patients with BPD. Our work contributes to a better understanding of alterations in interpersonal trust and especially their possible origins. Specifically, our findings encourage addressing difficulties to trust close others such as family members or romantic partners, omniscient negative memory and interpretation biases.

## Data Availability

The datasets generated during and/or analyzed during the current study are available from the corresponding author on reasonable request. The datasets generated and analyzed during the current study are not publicly available to maintain confidentiality of our participants.
